# Evaluating the Potential of Three Sperm Surface Antigens as Egg-adhesion Biomarkers for Human Sperm Selection

**Published:** 2018

**Authors:** Mahnaz Heidari, Sara Darbandi, Mahsa Darbani, Naser Amirjanati, Mahmood Bozorgmehr, Hojjat Zeraati, Mohammad Mehdi Akhondi, Mohammad Reza Sadeghi

**Affiliations:** 1- Reproductive Biotechnology Research Center, Avicenna Research Institute, ACECR, Tehran, Iran; 2- Department of Andrology, Avicenna Fertility Clinic, Tehran, Iran; 3- Monoclonal Antibody Research Center, Avicenna Research Institute, ACERCR, Tehran, Iran; 4- Department of Epidemiology and Biostatistics, Tehran University of Medical Sciences, Tehran, Iran

**Keywords:** DJ-1, Heat shock protein A2, Serum amyloid P component, Sperm DNA

## Abstract

**Background:**

The selection of sperm with good genomic integrity and surface antigens is suggested for improving assisted reproductive technology (ART) outcome. The aim of this study was evaluating the heat shock protein (HSPA2), Dj-1 and serum amyloid P compound (SAP) three sperm surface proteomes as biomarkers for this purpose.

**Methods:**

In this study, semen samples were obtained from 114 men who presented at Avicenna Fertility Clinic for their treatment. The semen characteristics, DNA fragmentation Index (DFI), chromatin maturation index (CMI), biomarker levels, and their embryo quality were considered. The paired-samples t-test and independent-samples t-test were used for analyzing the data and p-values<0.05 were considered significant.

**Results:**

Outcomes exhibited the major reduction in HSPA2, DJ-1 and SAP following reduction in sperm quality and DNA integrity (p<0.001) with cut-off value of 14% (HSPA2), 12% (DJ-1) and 10% (SAP). The specificity of these three biomarkers was 95.2, 73.8 and 88.1%, respectively. Also, DFI (p<0.001), CMI (p<0.05), cleavage (p<0.05), and embryos quality (p<0.001) decreased significantly in abnormal spermiogram (ANS) group in compared with normal spermiogram (NS) group. It was shown that DFI was 97.1% in HSPA2, 76.5% in DJ-1 and 94.1% in SAP, and CMI was 95.0%, 75.50% and 87.5%, respectively. The significant correlation was found between of the three biomarkers and CMI (p<0.001), DFI (p<0.001) and embryos quality (p<0.001).

**Conclusion:**

By comparing the efficiency of these three biomarkers for selecting sperm with the lowest level of chromatin damages, it seems that selection based on HSPA2 has significance over others.

## Introduction

Condensing the sperm DNA over 10-fold has a vital role in keeping paternal DNA integrity compared with somatic cell nuclei (1). Studies proved that sperm DNA integrity is linked with sperm quality and male fertility (2). Furher-more, a strong reverse relation has been found between sperm DNA fragmentation and the rate of clinical pregnancy in ARTs (3). Accordingly, more advanced techniques are required to evaluate the optimal quality of sperm and recognize the functional sperm from malfunctioning ones. While sperm preparation in ARTs with density gradient centrifugation (DGC) has been thought to result in enrichment of sperm with intact chromatin, based on our knowledge, till now there is no reliable method for the clinical selection of sperm based on its DNA integrity and chromatin maturity and more improvements are necessary to progress it (3–6). Sperm-membrane proteins have a direct role in sperm-egg adhesion and fusion through fertilization (7). Many sperm selection methods exist based on sperm surface characteristics including electrophoresis, zeta potential, hyaluronic acid (HA), Annexine-V sperm sorting by magnetic activated cell sorting (MACs) and flow cytometer (FCM) these methods are applied at clinical level for diagnosis and treatment of severe male infertility particularly in cases of repeated implantation failure (RIF) (8–11). The selection of sperm with good genomic integrity and surface antigens is suggested for improving assisted reproductive technology (ART) outcome (12–16). Some of the main potential biomarkers involved in zona pellucida penetration, sperm binding and fertilization of oocyte, are heat shock protein (HSPA2), serum amyloid P compound (SAP), cysteine-rich secretory proteins (CRISP), fertilin β (Fβ), PH-20, DJ-1 and epididymis P34H protein (17–21). Presence of these molecules on the sperm surface and separation of sperm based on them depend on the accessibility of a specific ligand for planning a commercial test. Specific antigens like HSPA2, DJ-1 and SAP, as potent ligands for finding, splitting-up and measuring other recommended biomarkers were used in different investigations (22–24). These three biomarkers are assimilated with the sperm membrane developing through late steps of spermatogenesis in the testis and sperm maturation in epididymis and they improve capacitation which is involved in fertilization (25, 26). Presence of their receptors on human oocyte shows an essential character in sperm-oocyte communication and fertilization process (27). Fixing the DNA strand breaks, replacement of protamine through nuclear compaction and eliminating the cytoplasm in the last stages of sperm maturation are the particular functions for these three proteins in human testis (28). These three biomarkers also express in the equatorial piece of the matured sperm head and/or the tail through spermatogenesis in the testis (29, 30). Moreover, DJ-1 has a main role in androgen receptor-dependent transcriptional activity and oxidative stress (31–35). SAP in human shows both physiological and pathological participation in inflammation, immunity and apoptosis (36). In this study, the cut-off values of HSPA2, DJ-1 and SAP and their potential for sperm selection in ARTs and also, the ability of each biomarker to separate sperm according to its DNA safety considerations and their embryo cleavage and quality were evaluated as well.

## Methods

### Semen collection and preparation:

Semen specimens were obtained from 114 men at Avicenna Fertility Clinic affiliated to Avicenna Research Institute (Iran, Tehran). This study was approved by the bioethics committees of Avicenna Research Institute (Tehran, Iran) and written informed consent was gained from each donor. All subjects had no history of radiotherapy, chemotherapy, taking chronic medicine or varicocele and semen samples were collected from men after 48–72 *hr* of sexual abstinence. Semen was permitted to liquefy at 37°*C* for 30 *min*. An aliquot of the sample was used for semen analysis and the mature sperm were separated by DGC method (300 *g* for 20 *min*) with PureSperm® (Nidacon International AB, Sweden). The sperm pellet was washed twice and aliquoted for using in following procedures. Semen parameters were measured by computer-aided sperm analysis (CASA) according to WHO guidelines (37). Sperm parameters were considered normal when sperm concentration was ≥15 *million/ml*, viability ≥58%, normal sperm morphology ≥4% and total sperm motility ≥40%. The specimens were classified according to normal and abnormal spermiogram into two groups of NS group with normal spermiogram and ANS group with abnormal spermiogram.

### Biomarker levels:

The presence of HSPA2, DJ-1 and SAP on sperm surface was compared in NS and ANS groups. Firstly, 1×10^6^ sperm were washed twice at 300 *g* for 10 *min* with FCM buffer (Ice-cold PBS pH=7.2, containing 1% goat serum and 2% FCS). Then, 100 *μl* of affinity purified rabbit antibody against biomarkers was added. Anti-HSPA2, anti-DJ-1 and anti-SAP (Avicenna Research Institute, Tehran, Iran) and incubated according to its protocol. Sperm were washed as described above and incubated with 100 *μl* FITC-conjugated goat anti-rabbit (Abcam, Germany), for 30 *min* at 4*°C*. In order to assess sperm viability, all sperm fractions were labelled with propidium iodide (PI) (Sigma, Germany). As a control, samples without any primary and secondary antibodies (Evaluation of auto-fluorescence) or without just primary antibodies (Negative control) were used. Ten thousand sperm were analyzed per sample with a flow rate of FCM (Partec PAS, Germany). The differences among individual samples in the percentage of sperm above the set threshold level of fluorescence intensity were assessed and statistically compared. The analysis was done by FlowJo 7.5.4 software (Tree Star, Ashland, USA) (22).

### Sperm DNA fragmentation index (DFI) and chromatin maturity index (CMI):

The percentage of abnormal DNA and mature chromatin was reported as DFI and CMI. Semen samples were washed with PBS and diluted to reach 1×10^6^ *sperm/ml* concentration. The assessment of DFI and CMI were respectively approved by sperm chromatin structure assay (SCSA) (38) and Chromomycin A3 staining assay (39) according to the protocols. The procedure of SCSA involved Acridine Orange (AO) (Sigma, Germany) staining of sperm and subsequent flow cytometric measurement (40). To perform the assay, semen samples were placed on liquid ice; all succeeding steps were performed at 4*°C*. Samples were diluted with TNE buffer (0.15 *mol/l* NaCl, 0.01 *mol/l* Tris, 0.001 *mol/l* EDTA, pH=7.4) to obtain the sperm concentration of <2×10^6^ *sperm/ml*. A 200-*μl* aliquot was removed and admixed with 400 *μl* of a low-pH detergent solution (0.15 *mol/l* NaCl, 0.08N HCl, 0.01% Triton X-100, pH=1.4). After 30 *s*, 1.2 *ml* of staining solution (6 *μg/ml* AO, chromatographically purified in 0.2 *mol/l* Na_2_HPO_4_, 1 *mmol/l* di-sodium EDTA, 0.15 *mol/l* NaCl, 0.1 *mol/l* citric acid monohydrate, pH=6.0) was added, and the stained sample was placed into the flow cytometer sample chamber. Abnormal chromatin structure, defined as an increased susceptibility to acid or heat-induced denaturation in situ, was quantitated by FCM measurement of the metachromatic shift from green (Native DNA) to red (Denatured, single-stranded DNA) fluorescence (Olympus, Tokyo, Japan). Final result was presented as DFI (%).

The pellets were processed with a fixed Carnoy’s solution for 5 *min* at 4*°C* and then were stained with 100 *μl* of 0.25 *mg/ml* CMA3 (Sigma, Germany) solution at room temperature. After flow cytometric based CMA3 staining assay, samples which were exposed to CMA3 were washed twice with PBS and assessed by Partec PAS flow cytometer, using an argon laser with an excitation wave length of 488 *nm*. Flow cytometer from Chromomycin A3 stained sperm was collected in fluorescence detector-2 (FL-3) with a 585/42 *nm* band pass filter. A minimum of 10000 sperm were examined for each assay and analyzed using flow-jo software. A positive control was obtained by pre-incubating the spermatozoa with 200 *mmol* dithiothreitol, a disulphide reducing agent, at 37*°C* for 10 *min*. The percentage of immature sperm chromatin was reported as CMI (%).

### Sperm viability and mitochondrial function assay:

Sperm viability was assessed by eosin-nigrosin assay according to WHO protocols by mixing approximately equal volumes of semen and stain (37). Sperm mitochondrial function was assessed by Rodamine123 (Rh123) (Sigma, Germany) staining based on its protocol (41).

Mitochondrial function was analyzed by FCM in Rodamine (Rh 123) stained sperm. Semen aliquot was washed and 10^6^/*ml* sperm were incubated in PBS containing 0.01 *mg/ml* Rodamine123 (R123) at 25°*C* for 10 *min* in dark place. The stained sperm were washed, centrifuged (300 *g* for 10 *min*) and incubated in PBS and immediately analyzed by FCM. The FCM analysis was performed using an argon laser at 488 *nm* for excitation (27).

### Fertilization and embryo quality:

Ovarian hyper-stimulation was done according to the long luteal suppression protocol which uses GnRHa and with a combination of human menopausal gonadotropin (hMG). Ovulation was triggered by the administration of human chorionic gonadotropin (hCG). Oocytes were collected 36 hrs post hCG, using a simple lumen aspiration needle. Oocytes were retrieved by transvaginal ultrasound guided follicle aspiration. The oocytes containing cumulus cells were collected from clear follicular fluid. Granulosa cells were detached from collected oocytes using enzymatic and mechanical digestion. For all samples, sperm were injected (ICSI) to metaphase II (MII) oocytes to evaluate fertilization rate and embryo quality. The fertilized oocytes were evaluated by the presence of two pronuclei (2PN) in fertilized oocytes 18 *hr* after the injection. Embryo quality was estimated by morphological principles documented according to the fragmentation degree and the regularity of blastomeres 48–72 *hr* post-ICSI technique. Briefly, the embryos were categorized as grade A (Lacking fragmentation), grade B (Fragmentation<20%), and grade C (Fragmentation>20%) based on their quality (42). The embryos were transferred either at 48 or 72 *hr* stage.

### Statistical analysis:

All statistical analysis was done by the statistical package for social sciences (SPSS) version 19 (SPSS Inc, USA). The K.S test was used for controlling normal distribution and normal data presented as mean±SD. The paired-samples t-test and independent-samples t-test were used for analyzing and data. The p-values of <0.05 were considered significant. Pearson’s correlations were used for parametric variables. For the calculation of a cut-off point, differences in the expression rates were designed and cross-tab was used.

## Results

The outcomes of all 114 semen samples are exhibited in table 1. With comparison of NS and ANS groups, it has been shown that the average male age (32.55±0.74 and 32.40±1.11, respectively in both groups), female age (31.38±0.73 and 32.40±0.56) and semen volume (4.37±1.04 and 4.94±1.76) had no statistically significant changes between two groups. But, the documents demonstrated that sperm concentration (p<0.001), progressive motility (p<0.001), normal morphology (p<0.001), DFI (p<0.001), CMI (p<0.001), viability (p<0.001), mitochondrial function (0.01), HSPA2 (p<0.001), DJ-1 (p<0.001) and SAP (p< 0.001) were meaningfully lower in ANS compared with NS. The fertilization and embryo features were considered through visualizing two-PN and cleavage stage in one, two and three days after ICSI. The outcome indicated that there were statistically significant changes in embryo cleavage phase (p<0.05) and grade A embryo quality (p<0.001) among these two groups (Table 1).

**Table 1.** The results of parameters evaluated between normal spermiogram (NS) and abnormal spermiogram (ANS) groups

**Parameter**	**NS (n=42)**	**ANS (n=74)**	**p-value**
**Concentration (×10^6^/*ml*)**	45.57±1.76	32.40±1.11	p<0.001
**Viability (%)**	81.43±1.24	77.46±1.02	p<0.001
**Mitochondrial function (%)**	77.45±1.69	66.76±1.07	0.016
**CMI (%)**	20.62±0.86	23.51±0.82	0.029
**DFI (%)**	21.10±1.196	30.42±1.78	p<0.001
**DJ-1 (%)**	16.77±0.94	12.32±0.81	p<0.001
**SAP (%)**	15.81±0.83	10.43±0.68	p<0.001
**HSPA2**	27.88±6.58	15.01±7.27	p<0.001
**Cleavage (%)**	75.05±21.02	62.30±26.69	0.020
**Grade A (%)**	49.27±25.87	29.09±32.01	0.001
**Grade B (%)**	14.52±14.78	12.30±16.45	0.470
**Grade C (%)**	1.65±5.26	8.70±15.69	0.010

Note: Values are reported by mean±SD, DNA fragmentation index (DFI), chromatin maturation index (CMI), grade A (Embryo without fragmentation), grade B (Embryo fragmentation<20%), and grade C (Embryo fragmentation >20%)

In addition, the outcome exhibited the significant decrease of HSPA2, DJ-1 and SAP with regard to cut off value of 14%, 12% and 10%, respectively following the reduction in sperm quality. The specificity of these three biomarkers was 95.2, 73.8 and 88.1 % in NS group. In addition, among the sperm with biomarker levels higher than cut off 97.1% with HSPA2, 76.5% with DJ-1 and 94.1% with SAP normal DNA integrity was observed, but normal chromatin maturation was observed with levels higher than cut offs 95.0% with HSPA2, 75.50% with DJ-1 and 87.5% with SAP. The sperm analyzed with these three approaches in ANS group showed that, 78.4% with HSPA2, 67.6% with DJ-1 and 70.3% with SAP had normal DNA integrity, but 59.6% with HSPA2, 39.7% with DJ-1 and 44.11% with SAP had normal chromatin maturation (Table 2). The probable influence of these three biomarker levels on sperm and embryo quality were analyzed and are manifested in table 3. The major correlation was detected in DJ-1 and SAP level in sperm population with CMI (p<0.001), DFI (p<0.001), and grade A embryo (p<0.001). Moreover, the results presented while these three biomarkers had altered in their levels in NS and ANS groups, HSPA2 meaningfully has greater level (p<0.001) compared with others. After evaluating the correlation, the important correlations were detected between HSPA2 level in sperm population and progressive motility (r=0.32, p<0.01), CMI (r=−0.63, p<0.001), DFI (r=−0.66, p<0.001), cleavage (r=0.33, p<0.01), grade A embryo (r=0.52, p<0.001) and grade B embryo (r=0.28, p<0.05) which is shown in table 3. Also, DJ-1 and SAP levels had correlation with CMI (r=−0.64, r=−0.65, respectively, p<0.001), DFI (r=−0.65, r=−0.67, p<0.001), and grade A embryo (r=0.40, r=0.40, p<0.001).

**Table 2.** The HSPA2, Dj-1 and SAP and the cut-off values in normal spermiogram (NS) and abnormal spermiogram (ANS) groups

**Groups**		**HSPA2**			**DJ-1**			**SAP**		

		**Cut off 14%**	**DFI**	**CMI**	**Cut off 12%**	**DFI**	**CMI**	**Cut off 10%**	**DFI**	**CMI**
**NS (n=42)**	Specificity (%)	95.2	97.1	95.0	73.8	76.5	75.50	88.1	94.1	87.5
	Sensitivity (%)	4.8	2.9	5.0	26.2	23.5	25.0	11.9	5.9	12.5
**ANS (n=72)**	Specificity (%)	48.6	78.4	59.6	43.1	67.6	39.7	48.6	70.3	44.11
	Sensitivity (%)	51.4	21.6	40.4	56.9	32.4	60.3	51.4	29.7	55.2

Note: DNA fragmentation index (DFI), chromatin maturation index (CMI)

**Table 3.** The correlation of HSPA2, Dj-1 and SAP with sperm parameters, fertilization and embryo quality

**Parameters**	**HSPA2**		**DJ-1**		**SAP**	

	**r**	**p-value**	**r**	**p-value**	**r**	**p-value**
**Concentration (×106/*ml*)**	0.05	0.656	0.17	0.164	0.19	0.180
**Progressive**	0.32	0.007^**^	0.14	0.232	0.13	0.283
**Morphology**	0.19	0.106	0.23	0.061	0.20	0.095
**Vitality**	0.08	0.509	0.008	0.94	0.29	0.011
**Mitochondria function**	0.28	0.173	0.11	0.360	0.09	0.00
**DFI**	−0.66	0.000 ^***^	−0.64	0.000 ^***^	−0.65	0.000 ^***^
**CMI**	−0.63	0.000 ^***^	−0.61	0.000 ^***^	−0.67	0.000 ^***^
**Cleavage**	0.31	0.008 ^**^	0.20	0.095	0.18	0.132
**Grade A**	0.52	0.000 ^***^	0.40	0.000 ^***^	0.40	0.000 ^***^
**Grade B**	0.28	0.020 ^*^	0.19	0.102	0.14	0.240

Note: r; indicates the Pearson correlation coefficient. The value of p<0.05 was considered significant. Sperm concentration (Concentration), DNA fragmentation index (DFI), chromatin maturation index (CMI), grade A (Embryo without fragmentation), and grade B (Embryo fragmentation<20%).

^*^, ^**^ and ^***^ means p<0.05, p<0.01, p<0.001, respectively

## Discussion

In this paper, the levels of HSPA2, Dj-1 and SAP were determined in sperm population in two groups of men with normal and abnormal spermiogram. In addition, the correlations of sperm chromatin maturity-integrity and fertilization rate with these biomarkers were considered. Furthermore, the cut-off value of three biomarkers was compared in relation with DFI, CMI and embryo quality to find potential biomarkers for sperm selection with the lowest level of chromatin damage. These findings exhibited that, regarding the alteration of these biomarker levels in sperm of men with normal and abnormal semen parameters in ANS group, the sperm had lower levels of HSPA2, Dj-1 and SAP and higher level of DNA damage and abnormal chromatin packaging in comparison with NS group. This outcome was in line with other studies illustrating the localization of HSPA2, Dj-1 and SAP as an important sperm-egg interaction factor in human sperm surface and the correlation of them with sperm normal morphology, concentration, DNA fragmentation and fertility potential (43–45). In this paper, DNA fragmentation levels were correlated with abnormal sperm parameters which was reported previously by others (46, 47), although some investigators showed this relation with normal semen parameters too (48). As these three biomarker levels were very different in sperm of men with normal and abnormal semen parameters, so a representative cut-off level for classifying characters and sperm based on these antigens appears to be an important step. As there was no transportation over the female reproductive tract in ICSI, a better understanding of the relationship between sperm DNA integrity and embryonic developmental potential is necessary. While modern semen analysis could predict male fertility, these parameters could not distinguish exact failings such as sperm immature chromatin and DNA damage. Accordingly, searches for identifying the conventional parameters to progress the predictability are required to find functional sperm from others (45). Sperm preparation for ARTs with differential DGC has been found to result in enhancement of sperm with intact chromatin, which in turn was likely to progress the risks of a successful clinical outcome (49). Whereas success rates were known to vary usually through clinics, more advances are necessary to develop it. Currently, new selecting sperm methods have been constructed on sperm molecular characterization and function. Selecting sperm binding to antibody for ICSI was thought to be one such advance. Numerous studies have presented that the sperm DNA damage was negatively correlated to pregnancy rate and embryo quality (50). The reduced level of these biomarkers from the human sperm led to a reduced power for sperm-egg recognition and fertilization following ARTs (12–16). This model was drawn considering the fact that these were taken in mature sperm and were preferably situated in the head area to be shared in oocyte (12, 13, 51). As indicated previously, there was no such standard in its place of routine sperm parameter test (Like morphology, count, motility, *etc*.), even though these parameters do not have the necessary and reliable efficacy to accurately evaluate male fertility. Based on the outcomes of the current study, reduction in these biomarkers might account as two characteristics of sperm in ANS men. Our results were in agreement to earlier studies that had separately investigated these parameters in infertile men (44, 52). Regarding the outcomes of the current study, the reduction of these three biomarkers clearly exhibited chromatin abnormalities in ANS samples as was shown previously by others (53, 54). By comparing the efficiency and cut off of these three biomarkers with the lowest level of chromatin damages, it seems that selection resulting in HSPA2 has significance over others. Major negative correlation between the HSPA2, Dj-1 and SAP level and the sperm DNA fragmentation, and chromatin maturation might be apparent due to its role in sperm protamination and suitable folding of chromatin during sperm maturation in testis manifests that these biomarkers might be a good choice for selecting the intact sperm for ARTs (55). Hence, it seems that fixing the DNA strand breaks, replacement of protamine during nuclear compaction and eliminating the cytoplasm during the last stages of sperm maturation were the specific functions that were planned for these three biomarkers in human testis. However, HSPA2 has also a crucial role in these functions (44, 56).

## Conclusion

In conclusion, it is suggested that while HSPA2, Dj-1 and SAP had the potential to be a method for selecting sperm with the lowest level of chromatin damages in ARTs, it seems that sperm selection based on HSPA2 level can be improved for this purpose. As HSPA2 is induced in response to today's environmental agents like stress, air pollution, and oxidative stress (57, 58), this method may be helpful for selecting sperm with the lowest damage of reactive oxygen and nitrogen species.

## References

[1] Seli E Sakkas D. Spermatozoal nuclear determinants of reproductive outcome: implications for ART. Hum Reprod Update. 2005;11(4):337–49. 15863434 10.1093/humupd/dmi011

[2] Rex AS Aagaard J Fedder J. DNA fragmentation in spermatozoa: a historical review. Andrology. 2017;5 (4):622–30. 28718529 10.1111/andr.12381 PMC5601286

[3] Smith R Kaune H Parodi D Madariaga M Morales I Rios R [Extent of sperm DNA damage in spermatozoa from men examined for infertility. Relationship with oxidative stress]. Rev Med Chil. 2007;135(3):279–86. Spanish. 17505572 10.4067/s0034-98872007000300001

[4] Evenson DP. The sperm chromatin structure assay (SCSA(®)) and other sperm DNA fragmentation tests for evaluation of sperm nuclear DNA integrity as related to fertility. Anim Reprod Sci. 2016;169: 56–75. 26919909 10.1016/j.anireprosci.2016.01.017

[5] Evenson DP Larson KL Jost LK. Sperm chromatin structure assay: its clinical use for detecting sperm DNA fragmentation in male infertility and comparisons with other techniques. J Androl. 2002;23(1):25–43. 11780920 10.1002/j.1939-4640.2002.tb02599.x

[6] Irez T Sahmay S Ocal P Goymen A Senol H Erol N Investigation of the association between the outcomes of sperm chromatin condensation and de-condensation tests, and assisted reproduction techniques. Andrologia. 2015;47(4):438–47. 24766543 10.1111/and.12286

[7] Naaby-Hansen S Herr JC. Heat shock proteins on the human sperm surface. J Reprod Immunol. 2010; 84(1):32–40. 19962198 10.1016/j.jri.2009.09.006 PMC2898571

[8] Berteli TS Da Broi MG Martins WP Ferriani RA Navarro PA. Magnetic-activated cell sorting before density gradient centrifugation improves recovery of high-quality spermatozoa. Andrology. 2017;5(4): 776–82. 28622434 10.1111/andr.12372

[9] Erberelli RF Salgado RM Pereira DH Wolff P. Hyaluronan-binding system for sperm selection enhances pregnancy rates in ICSI cycles associated with male factor infertility. JBRA Assist Reprod. 2017;21(1):2–6. 28333023 10.5935/1518-0557.20170002 PMC5365191

[10] Razavi S Nasr-Esfahani M Deemeh M Shayesteh M Tavalaee M. Evaluation of zeta and HA-binding methods for selection of spermatozoa with normal morphology, protamine content and DNA integrity. Andrologia. 2010;42(1):13–9. 20078511 10.1111/j.1439-0272.2009.00948.x

[11] Simon L Ge SQ Carrell DT. Sperm selection based on electrostatic charge. Methods Mol Biol. 2013; 927:269–78. 22992922 10.1007/978-1-62703-038-0_25

[12] Nixon B Bromfield EG Dun MD Redgrove KA McLaughlin EA Aitken RJ. The role of the molecular chaperone heat shock protein A2 (HSPA2) in regulating human sperm-egg recognition. Asian J Androl. 2015;17(4):568–73. 25865850 10.4103/1008-682X.151395 PMC4492046

[13] Redgrove KA Nixon B Baker MA Hetherington L Baker G Liu DY The molecular chaperone HSPA2 plays a key role in regulating the expression of sperm surface receptors that mediate sperm-egg recognition. PLoS One. 2012;7(11): e50851. 23209833 10.1371/journal.pone.0050851 PMC3510172

[14] Bromfield EG Nixon B. The function of chaperone proteins in the assemblage of protein complexes involved in gamete adhesion and fusion processes. Reproduction. 2013;145(2):R31–42. 23166368 10.1530/REP-12-0316

[15] Martinez-Leon E Osycka-Salut C Signorelli J Pozo P Perez B Kong M Fibronectin stimulates human sperm capacitation through the cyclic AMP/protein kinase A pathway. Hum Reprod. 2015;30(9):2138–51. 26109618 10.1093/humrep/dev154

[16] Cho C. Testicular and epididymal ADAMs: expression and function during fertilization. Nat Rev Urol. 2012;9(10):550–60. 22926424 10.1038/nrurol.2012.167

[17] Fusi F Bronson RA. Sperm surface fibronectin expression following capacitation. J Androl. 1992;13 (1):28–35. 1551804

[18] Bohring C Krause W. Characterization of spermatozoa surface antigens by antisperm antibodies and its influence on acrosomal exocytosis. Am J Reprod Immunol. 2003;50(5):411–9. 14750700 10.1034/j.1600-0897.2003.00103.x

[19] Kiernan UA Nedelkov D Tubbs KA Niederkofler EE Nelson RW. Proteomic characterization of novel serum amyloid P component variants from human plasma and urine. Proteomics. 2004;4(6): 1825–9. 15174148 10.1002/pmic.200300690

[20] Snell WJ White JM. The molecules of mammalian fertilization. Cell. 1996;85(5):629–37. 8646772 10.1016/s0092-8674(00)81230-1

[21] Wagenfeld A Yeung CH Shivaji S Sundareswaran VR Ariga H Cooper TG. Expression and cellular localization of contraception-associated protein. J Androl. 2000;21(6):954–63. 11105923

[22] Capkova J Kubatova A Ded L Tepla O Peknicova J. Evaluation of the expression of sperm proteins in normozoospermic and asthenozoospermic men using monoclonal antibodies. Asian J Androl. 2016;18(1):108–13. 25926605 10.4103/1008-682X.151400 PMC4736337

[23] Kaya A Memili E. Sperm macromolecules associated with bull fertility. Anim Reprod Sci. 2016; 169:88–94. 26925808 10.1016/j.anireprosci.2016.02.015

[24] Gorner K Holtorf E Odoy S Nuscher B Yamamoto A Regula JT Differential effects of Parkinson's disease-associated mutations on stability and folding of DJ-1. J Biol Chem. 2004;279(8): 6943–51. 14607841 10.1074/jbc.M309204200

[25] Agarwal A Said TM. Role of sperm chromatin abnormalities and DNA damage in male infertility. Hum Reprod Update. 2003;9(4):331–45. 12926527 10.1093/humupd/dmg027

[26] Sun Y Zhang WJ Zhao X Yuan RP Jiang H Pu XP. PARK7 protein translocating into spermatozoa mitochondria in Chinese asthenozoospermia. Reproduction. 2014;148(3):249–57. 24920663 10.1530/REP-14-0222

[27] Kaji K Kudo A. The mechanism of sperm-oocyte fusion in mammals. Reproduction. 2004;127(4): 423–9. 15047933 10.1530/rep.1.00163

[28] An CN Jiang H Wang Q Yuan RP Liu JM Shi WL Down-regulation of DJ-1 protein in the ejaculated spermatozoa from Chinese asthenozoospermia patients. Fertil Steril. 2011;96(1):19–23.e2. 21575935 10.1016/j.fertnstert.2011.04.048

[29] Barrier Battut I Kempfer A Becker J Lebailly L Camugli S Chevrier L. Development of a new fertility prediction model for stallion semen, including flow cytometry. Theriogenology. 2016;86(4):1111–31. 27207472 10.1016/j.theriogenology.2016.04.001

[30] Widlak W Vydra N. The role of heat shock factors in mammalian spermatogenesis. Adv Anat Embryol Cell Biol. 2017;222:45–65. 28389750 10.1007/978-3-319-51409-3_3

[31] Saylan A Duman S. Efficacy of Hyaluronic Acid in The Selection of Human Spermatozoa with Intact DNA by The Swim-up Method. Cell J. 2016; 18(1):83–8. 27054122 10.22074/cellj.2016.3990 PMC4819390

[32] Bonifati V Rizzu P Van Baren MJ Schaap O Breedveld GJ Krieger E Mutations in the DJ-1 gene associated with autosomal recessive early-onset parkinsonism. Science. 2003;299(5604): 256–9. 12446870 10.1126/science.1077209

[33] Mitsumoto A Nakagawa Y. DJ-1 is an indicator for endogenous reactive oxygen species elicited by endotoxin. Free Radic Res. 2001;35(6):885–93. 11811539 10.1080/10715760100301381

[34] Taira T Saito Y Niki T Iguchi-Ariga SM Takahashi K Ariga H. DJ-1 has a role in antioxidative stress to prevent cell death. EMBO Rep. 2004;5(2): 213–8. 14749723 10.1038/sj.embor.7400074 PMC1298985

[35] Takahashi K Taira T Niki T Seino C Iguchi-Ariga SM Ariga H. DJ-1 positively regulates the androgen receptor by impairing the binding of PIASx alpha to the receptor. J Biol Chem. 2001; 276(40):37556–63. 11477070 10.1074/jbc.M101730200

[36] Bickerstaff M Botto M Hutchinson W Herbert J Tennent G Bybee A Serum amyloid P component controls chromatin degradation and prevents antinuclear autoimmunity. Nat Med. 1999;5 (6):694–7. 10371509 10.1038/9544

[37] World Health Organization WHO laboratory manual for the examination and processing of human semen. 5th ed Geneva: World Health Organization 2010 286 p.

[38] Torki-Boldaji B Tavalaee M Bahadorani M Nasr-Esfahani MH. Selection of physiological spermatozoa during intracytoplasmic sperm injection. Andrologia. 2017;49(1). 10.1111/and.12579 27037571

[39] Shamsi MB Imam SN Dada R. Sperm DNA integrity assays: diagnostic and prognostic challenges and implications in management of infertility. J Assist Reprod Genet. 2011;28(11):1073–85. 21904910 10.1007/s10815-011-9631-8 PMC3224170

[40] Evenson DP Larson KL Jost LK. Sperm chromatin structure assay: its clinical use for detecting sperm DNA fragmentation in male infertility and comparisons with other techniques. J Androl. 2002; 23(1):25–43. 11780920 10.1002/j.1939-4640.2002.tb02599.x

[41] Wu YM Xia XY Pan LJ Shao Y Jin BF Huang YF [Evaluation of sperm mitochondrial function using Rh123/PI dual fluorescent staining]. Zhonghua Nan Ke Xue. 2006;12(9):803–6. Chinese. 17009532

[42] Kim JY Kim JH Jee BC Lee JR Suh CS Kim SH. Can intracytoplasmic sperm injection prevent total fertilization failure and enhance embryo quality in patients with non-male factor infertility? Eur J Obstet Gynecol Reprod Biol. 2014;178:188–91. 24793928 10.1016/j.ejogrb.2014.03.044

[43] Spinaci M Volpe S Bernardini C De Ambrogi M Tamanini C Seren E Immunolocalization of heat shock protein 70 (Hsp 70) in boar spermatozoa and its role during fertilization. Mol Reprod Dev. 2005;72(4):534–41. 16142794 10.1002/mrd.20367

[44] Motiei M Tavalaee M Rabiei F Hajihosseini R Nasr-Esfahani MH. Evaluation of HSPA2 in fertile and infertile individuals. Andrologia. 2013;45(1): 66–72. 22670834 10.1111/j.1439-0272.2012.01315.x

[45] Greco E Scarselli F Iacobelli M Rienzi L Ubaldi F Ferrero S Efficient treatment of infertility due to sperm DNA damage by ICSI with testicular spermatozoa. Hum Reprod. 2005;20(1):226–30. 15539441 10.1093/humrep/deh590

[46] Henkel R Schreiber G Sturmhoefel A Hipler UC Zermann DH Menkveld R. Comparison of three staining methods for the morphological evaluation of human spermatozoa. Fertil Steril. 2008;89(2): 449–55. 17601601 10.1016/j.fertnstert.2007.03.027

[47] Silva P Gadella B. Detection of damage in mammalian sperm cells. Theriogenology. 2006;65(5): 958–78. 16242762 10.1016/j.theriogenology.2005.09.010

[48] Boushaba S Belaaloui G. Sperm DNA fragmentation and standard semen parameters in algerian in fertile male partners. World J Mens Health. 2015; 33(1):1–7. 25927056 10.5534/wjmh.2015.33.1.1 PMC4412002

[49] Sauer R Coulam CB Jeyendran RS. Chromatin intact human sperm recovery is higher following glass wool column filtration as compared with density gradient centrifugation. Andrologia. 2012;44 Suppl 1:248–51. 21689135 10.1111/j.1439-0272.2011.01171.x

[50] Zini A Boman JM Belzile E Ciampi A. Sperm DNA damage is associated with an increased risk of pregnancy loss after IVF and ICSI: systematic review and meta-analysis. Hum Reprod. 2008;23 (12):2663–8. 18757447 10.1093/humrep/den321

[51] Liu F Qiu Y Zou Y Deng ZH Yang H Liu DY. Use of zona pellucida–bound sperm for intracytoplasmic sperm injection produces higher embryo quality and implantation than conventional intracytoplasmic sperm injection. Fertil Steril. 2011;95 (2):815–8. 20971463 10.1016/j.fertnstert.2010.09.015

[52] Ni K Spiess AN Schuppe HC Steger K. The impact of sperm protamine deficiency and sperm DNA damage on human male fertility: a systematic review and meta-analysis. Andrology. 2016;4(5): 789–99. 27231200 10.1111/andr.12216

[53] Bromfield E Aitken RJ Nixon B. Novel characterization of the HSPA2-stabilizing protein BAG6 in human spermatozoa. Mol Hum Reprod. 2015; 21(10):755–69. 26153132 10.1093/molehr/gav041

[54] Erata GO Kocak Toker N Durlanik O Kadioglu A Aktan G Aykac Toker G. The role of heat shock protein 70 (Hsp 70) in male infertility: is it a line of defense against sperm DNA fragmentation? Fertil Steril. 2008;90(2):322–7. 17880957 10.1016/j.fertnstert.2007.06.021

[55] Oliva R Castillo J. Proteomics and the genetics of sperm chromatin condensation. Asian J Androl. 2011;13(1):24–30. 21042303 10.1038/aja.2010.65 PMC3739392

[56] Nixon B Bromfield EG Cui J De Iuliis GN. Heat shock protein A2 (HSPA2): regulatory roles in germ cell development and sperm function. Adv Anat Embryol Cell Biol. 2017;222:67–93. 28389751 10.1007/978-3-319-51409-3_4

[57] Whitley D Goldberg SP Jordan WD. Heat shock proteins: a review of the molecular chaperones. J Vasc Surg. 1999;29(4):748–51. 10194511 10.1016/s0741-5214(99)70329-0

[58] Hahn GM Li GC. Thermotolerance and heat shock proteins in mammalian cells. Radiat Res. 1982;92 (3):452–7. 7178414

